# Burnout among Chinese EFL university instructors: a mixed-methods exploration of school climate, job demands, and emotion regulation

**DOI:** 10.3389/fpsyg.2025.1549466

**Published:** 2025-06-19

**Authors:** Lvliang Li

**Affiliations:** ^1^Department of Foreign Languages, Taiyuan Institute of Technology, Taiyuan, China; ^2^School of Academics, Applied Arts and Tourism, College of the North Atlantic, Stephenville, TX, Canada; ^3^School of Management, Shinawatra University, Pathum Thani, Thailand

**Keywords:** teacher burnout, school climate, job demands, emotion regulation, EFL instructors, Chinese universities, mixed-methods, higher education

## Abstract

**Introduction:**

Teacher burnout is a significant global concern in higher education, impacting instructor well-being and educational quality. English as a Foreign Language (EFL) instructors in Chinese universities face unique pressures that may heighten their burnout vulnerability. This mixed-methods study, guided by the Job Demands-Resources (JD-R) model, investigated the intricate relationships between perceived school climate, challenging job demands, emotion regulation, and teacher burnout among Chinese EFL university instructors.

**Methods:**

The study employed an explanatory sequential mixed-methods design. Quantitative data were collected from 478 Chinese EFL university instructors using scales assessing perceived school climate, challenging job demands, emotion regulation, and burnout; these data were analyzed using confirmatory factor analysis and structural equation modeling. Subsequently, qualitative data were gathered through semi-structured interviews with 21 instructors, selected purposively from the quantitative sample, and analyzed using thematic analysis to provide deeper insights.

**Results:**

Quantitative analysis revealed that a positive perceived school climate was associated with lower burnout, while high challenging job demands were associated with higher burnout. Emotion regulation significantly mediated these relationships, buffering the negative effects of job demands and enhancing the positive effects of school climate. The qualitative analysis yielded three key themes: (1) The Supportive yet Stifling School Climate, which highlighted appreciation for collegiality alongside constraints from rigidity and hierarchy; (2) The Weight of Unrealistic Expectations, detailing heavy workloads and competing demands; and (3) Navigating the Emotional Landscape, describing instructors’ strategies and challenges in managing emotions. These themes provided rich context, illustrating how instructors navigate institutional structures, workloads, and emotional stressors.

**Discussion:**

The findings underscore that both work environment characteristics (school climate and job demands) and personal resources (emotion regulation) are crucial in understanding EFL teacher burnout in the Chinese university context. The integrated results highlight the importance of fostering positive, supportive school climates that promote autonomy and recognize teaching excellence, alongside providing resources and support to help instructors manage job demands and enhance their emotion regulation skills.

## 1 Introduction

Instructors are widely recognized as crucial figures in students’ academic success (Murphy et al., 2004). However, the teaching profession is often fraught with worry, tension, and trepidation, which can impact instructors’ performance and well-being (Jeon et al., 2022; Montgomery and Rupp, 2005). Teachers are entrusted with a multitude of responsibilities, including classroom management, lesson planning, instructional delivery, and fostering student growth (Bottiani et al., 2019; Derakhshan et al., 2024; Wu and Zeng, 2024, 2025). Among the challenges faced by teachers is the risk of burnout, a syndrome characterized by emotional exhaustion, depersonalization, and reduced personal accomplishment (Maslach, 2003). Burnout can have detrimental effects on teachers’ mental health (Foley and Murphy, 2015; Herman et al., 2018; Wu et al., 2024a; Wu et al., 2024b), which in turn can impact the emotional climate of the classroom and students’ learning experiences (Braun et al., 2020; Chang, 2013; Vesely et al., 2013).

Several factors contribute to teacher burnout, including school climate, job demands, and emotion regulation. School climate, encompassing the attitudes, beliefs, and values shared within a school community (Dutta and Sahney, 2022; Mitchell et al., 2010), plays a crucial role in shaping teachers’ perceptions and experiences (Howard et al., 1987; Higgins-D’Alessandro, 2011). A positive school climate can foster a sense of belonging, reduce stress, and enhance job satisfaction (Skaalvik and Skaalvik, 2017; Ware and Kitsantas, 2007; Malinen and Savolainen, 2016). Job demands, referring to the physical, psychological, and cognitive demands of a job (Bakker and Demerouti, 2007; Galanakis and Tsitouri, 2022), can also significantly impact teacher well-being (Fernet et al., 2015). High job demands, coupled with inadequate resources, can lead to stress, exhaustion, and burnout (Alarcon, 2011; Han et al., 2020; Schaufeli and Bakker, 2004; Hobfoll, 2002). Furthermore, the ability to regulate emotions is essential for teachers to effectively manage the challenges and stressors inherent in their profession (Deng et al., 2022; Hu et al., 2023), with research underscoring its particular significance in the L2/EFL teaching context (Talbot and Mercer, 2018; Wu et al., 2023). Emotion regulation, defined as the processes involved in monitoring, evaluating, and modifying emotional reactions (Chang and Taxer, 2021), can significantly impact teachers’ well-being and their effectiveness in the classroom.

Despite the growing body of research on teacher burnout, there remains a need for comprehensive investigations that examine the interplay between school climate, job demands, emotion regulation, and burnout, particularly within the specific context of Chinese EFL university instructors. This mixed-methods study aims to address this gap by exploring the complex dynamics between these factors, utilizing both quantitative and qualitative approaches to gain a deeper understanding of the phenomenon. Specifically, this study investigates the mediating role of emotion regulation in the relationships between perceived school climate, challenging job demands, and teacher burnout among Chinese EFL instructors. By employing a mixed-methods design, this study not only examines the quantitative relationships between these variables but also delves into the lived experiences of EFL instructors through in-depth qualitative interviews. This approach allows for a more nuanced and holistic understanding of the factors contributing to teacher burnout in this unique context.

This study makes several important contributions to the field. First, it provides a detailed exploration of the mechanisms through which perceived school climate and job demands influence burnout, with a particular focus on the mediating role of emotion regulation. Second, it adopts a context-specific approach, shedding light on the unique dynamics within Chinese higher education that may contribute to teacher stress and burnout. Specifically, the Chinese higher education system features considerable diversity and is often characterized by a tiered structure reflecting variations in institutional prestige, research funding, and administrative level (e.g., Zhang and Wang, 2024b). Common distinctions include elite national ‘Double First-Class’ universities (Liu et al., 2019), numerous general provincial or ministerial universities, and other regular undergraduate institutions. These tiers often entail significant differences in available resources, research versus teaching expectations, student characteristics, and overall institutional climate, making it crucial to consider this diversity when studying instructor experiences. Finally, by incorporating qualitative data, this study provides rich insights into the lived experiences of EFL instructors, giving voice to their perspectives and illuminating the complexities of their emotional lives in relation to their work.

The findings of this study have significant implications for educational institutions, policymakers, and teacher support programs, offering valuable guidance for developing targeted interventions and support systems to enhance the well-being and job satisfaction of EFL instructors in China. By shedding light on the complex interplay between school climate, job demands, emotion regulation, and burnout, this study contributes to a deeper understanding of teacher well-being and offers practical solutions for creating a more supportive and fulfilling work environment for EFL instructors.

## 2 Literature review

### 2.1 Teacher burnout

Burnout in the teaching profession is a well-documented syndrome characterized by emotional exhaustion, diminished personal accomplishment, depersonalization, and decreased enthusiasm and commitment to success (Maslach, 1976). Teaching, in particular, has been associated with a high risk of burnout across various educational levels (e.g., Hakanen et al., 2006), and this phenomenon is increasingly studied within the specific context of EFL instruction in China. Recent investigations confirm that Chinese EFL instructors in higher education report experiencing moderately high levels of stress and burnout (Liu and Du, 2024), issues which may precipitate negative mental health outcomes and intentions to leave the profession (An and Tao, 2024).

The factors contributing to teacher burnout are multifaceted and often categorized into interpersonal, administrative, and personal domains (Chang, 2009). Interpersonal factors involve interactions with students and colleagues, while administrative factors include workload and classroom conditions. Personal factors like demographics can also play a role (Chang, 2013; Kara, 2020), and burnout negatively impacts job fulfillment (Kim et al., 2019; Nicuță et al., 2022; Zhu et al., 2018) and mental health (Jeon et al., 2022; Mérida-López and Extremera, 2017). Beyond these general factors, research focused on Chinese EFL university instructors highlights the critical role of personal psychological resources in mitigating burnout. Notably, teacher self-efficacy, or the belief in one’s own teaching capabilities, emerges as a particularly strong negative predictor of burnout, potentially explaining a large proportion of its variance (An and Tao, 2024; Li, 2023). Similarly, psychological capital (PsyCap)—encompassing hope, self-efficacy, resilience, and optimism—shows a direct negative relationship with burnout (Fathi et al., 2024; Liu and Du, 2024). Teacher resilience independently predicts lower burnout levels (Li, 2023), and mindfulness also appears protective, mediating the link between PsyCap and burnout; higher PsyCap fosters greater mindfulness, which in turn reduces burnout (Liu and Du, 2024). Furthermore, teacher well-being itself acts as a significant buffer against burnout, although perhaps secondary to the influence of self-efficacy (An and Tao, 2024). These findings collectively underscore the importance of internal strengths and positive psychological states for EFL teachers navigating demanding work environments in this specific context. Engaging in social activities and experiencing positive emotions have also been found generally to buffer against burnout (Herman et al., 2018).

While previous research has extensively explored various factors contributing to teacher burnout, and studies have increasingly recognized the importance of emotion regulation (e.g., Atmaca et al., 2020; Chang et al., 2022; Mulyani et al., 2021), further investigation is needed into its precise role and interplay with contextual factors within specific populations. The context of EFL instruction in Chinese universities presents such a case, where instructors may face a unique constellation of stressors potentially elevating burnout risk (Ma and Liu, 2024). Beyond general teaching demands, these educators often navigate distinct pressures concerning personal language proficiency maintenance, pedagogical adaptation to diverse student backgrounds and cultural contexts, and institutional targets related to internationalization and standardized testing. Moreover, the emotional labor involved in managing student language anxiety and facilitating cross-cultural communication can be particularly taxing for this group (Li and Liu, 2021; Morris and King, 2018). Understanding the complex relationships between work characteristics (school climate, job demands) and burnout requires considering how instructors cope amidst these specific contextual pressures. Therefore, examining the potential mediating function of emotion regulation within this distinct population is essential for developing targeted interventions that enhance well-being and support retention among Chinese university EFL educators. This study undertakes this investigation, aiming to identify mechanisms underlying burnout in this context and provide insights for evidence-based practices.

### 2.2 School climate

School climate is a complex and multifaceted concept that has garnered significant attention in educational research for decades (Aldridge and McChesney, 2018; Grazia and Molinari, 2021; Zhang et al., 2023). While its precise definition remains a matter of debate, it generally refers to the overall quality and character of the school environment, encompassing the social, emotional, and academic dimensions that shape interactions among students, teachers, and administrators (Cohen et al., 2009; National School Climate Center, 2007; Malinen and Savolainen, 2016; Mitchell et al., 2010; Zysberg and Schwabsky, 2021). Despite the challenges in precisely defining and measuring school climate, it is widely recognized as a crucial area of study for educational research and school improvement efforts.

Empirical studies have consistently demonstrated the profound impact of a positive school climate on various important educational outcomes (Tomaszewski et al., 2024). Students in schools characterized by a supportive and conducive climate tend to achieve higher levels of academic success, display greater enthusiasm for learning, exhibit reduced aggression, and experience lower detention rates (Boykin et al., 2024; Cohen et al., 2009; Gray et al., 2017; Thapa et al., 2013). Moreover, research indicates that a positive school climate also significantly influences teachers’ experiences and well-being (Cohen et al., 2009; Yang et al., 2022). Educators working in schools with a positive climate report higher levels of teaching efficacy, job satisfaction, and lower feelings of stress (Yao et al., 2015). Furthermore, recent studies specifically demonstrate that a supportive work environment, a key component of positive school climate, directly impacts the emotional experiences of L2 teachers (Zeng et al., 2024). Specifically, within the Chinese EFL university context, the work environment significantly shapes teachers’ experiences and outcomes. Supportive work environments and positive perceived climates are linked to beneficial outcomes such as more positive teacher achievement emotions (enjoyment, pride) and reduced negative emotions (anxiety, anger), which in turn foster teaching for creativity (Greenier et al., 2023; Wu and Zeng, 2024). Perceived climate, along with peer group interaction, directly impacts teaching for creativity, although the supervisory relationship may play a less significant role in this specific outcome (Ma and Wang, 2024). Aspects of support, such as available teaching resources and perceived autonomy, are also linked to higher teaching satisfaction, mediated by online teaching efficacy in digital environments (Han et al., 2021). Additionally, teachers’ perceptions of the *classroom* climate are crucial; positive classroom atmospheres are perceived by Chinese EFL teachers as preventing emotional exhaustion and potential job attrition, whereas negative climates are seen to exacerbate these issues (Mai, 2025). This highlights the interplay between the broader institutional environment and the immediate teaching context in influencing EFL teacher exhaustion and retention.

The value of improving school climate is increasingly recognized by policymakers and funding bodies globally (e.g., U.S. Department of Education, 2014). Beyond its direct impact on teacher well-being, school climate is vital in shaping the overall school culture; it fosters a sense of belonging and connectedness among students, teachers, and staff (Yao et al., 2015). A positive and inclusive climate promotes a supportive social environment, encouraging student engagement and constructive interpersonal relationships (Dutta and Sahney, 2022; Gray et al., 2017). When students feel valued and respected, they are more likely to develop a stronger sense of identity and commitment to their educational goals (Ibrahim and El Zaatari, 2020). Moreover, such nurturing climates contribute to safer learning environments by reducing incidents of bullying and disruptive behavior (Kutsyuruba et al., 2015; Wang, 2009).

Considering the broad impact of school climate, investigating it within the specific context of university EFL educators is therefore essential. This study explores the relationships between perceived school climate, challenging job demands, and teacher burnout among EFL instructors to better understand the pathways influencing teacher well-being in this higher education setting. Identifying key factors and mechanisms can help educational institutions and policymakers develop targeted, evidence-based interventions, ultimately fostering positive climates that benefit instructor well-being and enhance the EFL student educational experience.

### 2.3 Job demands-resources model as the framework

The theoretical underpinning for this investigation is the Job Demands-Resources (JD-R) model (Bakker and Demerouti, 2007; Demerouti and Bakker, 2011). The JD-R model provides a robust framework for understanding employee well-being across various occupations, including teaching, by proposing that well-being outcomes like burnout or engagement arise from the balance between two distinct categories of work characteristics: job demands and job resources (Lesener et al., 2019).

Job demands encompass the aspects of a job that require sustained physical, cognitive, or emotional effort and are thus associated with physiological and psychological costs (Bakker and Demerouti, 2017; Demerouti and Bakker, 2011). Examples relevant to the university teaching context include heavy workloads, time pressure, research demands, administrative duties, and emotionally taxing interactions with students or colleagues. The JD-R model outlines a *health impairment process*, suggesting that excessive or poorly managed job demands deplete an individual’s energy reserves over time, leading to strain, exhaustion, and ultimately burnout (Schaufeli and Bakker, 2004; Tummers and Bakker, 2021). In this study, the ‘challenging job demands’ experienced by Chinese EFL university instructors (such as teaching load, new challenges, and research demands) are conceptualized as specific job demands within this process, predicted to positively correlate with teacher burnout.

Conversely, job resources refer to those physical, psychological, social, or organizational aspects of the job that help individuals achieve work goals, reduce the costs associated with job demands, and stimulate personal growth, learning, and development (Bakker and Demerouti, 2007; Taris and Schaufeli, 2016). Job resources can operate at various levels, including organizational (e.g., autonomy, clarity of roles, opportunities for professional development), social (e.g., supervisor support, collegial relationships, positive feedback), and task levels (e.g., skill variety, task significance). The model also describes a *motivational process*, whereby the availability of job resources fosters work engagement, reduces cynicism, enhances job satisfaction, and promotes positive work attitudes (Bakker and Demerouti, 2017). Within the framework of this study, ‘perceived school climate’ is conceptualized primarily as a crucial set of job resources. Elements of a positive school climate, such as collaboration, supportive student relations, adequate school resources, shared decision-making, and encouragement of instructional innovation (Johnson et al., 2007), are expected to function as resources that buffer the negative effects of job demands and contribute positively to teacher well-being, thereby reducing burnout.

While the original JD-R model focused mainly on characteristics of the job environment, later extensions explicitly incorporate the role of *personal resources* (Xanthopoulou et al., 2007). Personal resources are individual psychological characteristics or capabilities that allow individuals to control and impact their environment effectively (Hobfoll, 2002). In this study, ‘emotion regulation’ is positioned as a critical personal resource interacting with the JD-R framework. Effective emotion regulation strategies are hypothesized to influence both the health impairment and motivational processes. By enabling instructors to manage the negative emotions arising from high job demands or challenging interactions, emotion regulation may mitigate the strain process. Furthermore, the ability to maintain a positive emotional state might help instructors better perceive, utilize, and benefit from available job resources (like a supportive climate), potentially enhancing the motivational process. Therefore, this study examines emotion regulation as a key mediating mechanism through which job demands (challenging tasks) and job resources (school climate) exert their influence on EFL teacher burnout.

Employing the JD-R model allows for a theoretically grounded exploration of how the specific job demands and resources within the Chinese EFL university context interact, and how personal resources like emotion regulation shape these dynamics, ultimately influencing instructors’ experiences of burnout.

### 2.4 Job demand-resource

Reflecting the utility of the framework described above, the Job Demand-Resource (JD-R) model has been widely investigated in the context of teachers’ engagement and burnout (De Carlo et al., 2019; Demerouti and Bakker, 2011; Han et al., 2020; Nicuță et al., 2022). For instance, Jansen in de Wal et al. (2020) explored the interplay between job resources, job demands, psychological needs fulfillment, and teachers’ commitment to professional development. They observed positive connections between job resource experience, dedication to professional growth, and self-motivation, while no significant relationship was found between demands and psychological needs satisfaction. Similarly, De Carlo et al. (2019) delved into the relationship between work–family conflict in instructors and job demands and resources. They discovered that job resources served as buffers, mitigating the impact of work–family conflict, and workload displayed a favorable connection with work–family conflict.

The utility of JD-R model extends beyond basic job characteristics, incorporating personal factors and exploring related constructs. For example, research has integrated individual characteristics within the JD-R framework, finding links between psychological demands or emotional repression and negative outcomes like anxiety and depression, while personal resources such as trust in colleagues or cognitive reappraisal relate positively to job satisfaction and excitement (Yin et al., 2018). Teacher self-efficacy has also been modeled as a key personal resource predicting motivation and disengagement (Vera et al., 2012). Studies further use the model to differentiate burnout from opposing constructs like work engagement (González-Romá et al., 2006) and related issues such as workaholism, which correlates with burnout but not necessarily engagement (Schaufeli et al., 2008). The framework also accommodates varying demands and resources across different career stages, illustrating how factors like economic concerns or caregiving demands can distinctly impact burnout and engagement over time (Salmela-Aro and Upadyaya, 2018).

Empirical applications consistently highlight the core JD-R pathways in educational settings, underpinning calls for systematic identification and management of demands and resources (Khan et al., 2014). Generally, studies report positive associations between job demands and burnout, alongside negative associations between job resources and burnout (Jagodics and Szabó, 2014), although occasional studies report complex or differing relationships in specific samples (e.g., Roslan et al., 2015). Resources like coworker support (Jagodics and Szabó, 2014) and broader organizational resources (Javed and Cheema, 2015) are often identified as important buffers or contributors to positive outcomes like work engagement. The model has proven applicable across various roles and contexts within education, revealing stress patterns among different types of university employees (Winefield et al., 2003) and proving useful for understanding school principals’ perceptions of demands and resources and their impact on exhaustion, satisfaction, and turnover intentions (Skaalvik, 2020).

Overall, extensive research confirms the value *of the* JD-R model in explaining how job demands and resources significantly influence outcomes like emotional exhaustion, job satisfaction, and motivation among educators. Understanding these core dynamics is crucial, particularly when considering the role of individual factors like emotion regulation in navigating the challenges *of the* teaching profession.

### 2.5 Emotion regulation

Emotion regulation encompasses a wide array of cognitive, behavioral, and physiological processes that enable individuals to effectively manage and respond to their emotional experiences (Gross, 2015; Gross and John, 2003; Gross and Thompson, 2007). It involves not only the control of emotional reactions but also the modification of emotional experiences to align with personal goals and social norms (Kolganov et al., 2022). Such regulation strategies are instrumental in shaping how individuals perceive, interpret, and express their emotions, ultimately influencing their emotional well-being and interactions with others (Li and Liu, 2021; Taylor et al., 2020).

Emotion regulation (ER) is highly relevant in educational settings, where teachers and students encounter diverse emotional situations (Braun et al., 2020; Chang et al., 2022; Zhang and Fathi, 2024; Jiang et al., 2016; Pishghadam and Sahebjam, 2012; Taxer and Gross, 2018). Effective ER by educators can foster a positive classroom climate (Atmaca et al., 2020; Chan, 2006; Taxer and Gross, 2018) and is crucial for their own well-being and effectiveness, potentially influencing student outcomes (Jiang et al., 2016). This capacity is particularly important for university EFL instructors in China. Research confirms a strong negative link between effective ER and burnout in foreign language teachers globally (Wang et al., 2024). For EFL instructors specifically, effective ER correlates not only with lower burnout but also with enhanced L2 grit, work engagement, and preferences for student-centered teaching styles (Fathi et al., 2024; Ma, 2023). Furthermore, ER strategies connect to broader mental health outcomes, potentially mediating links between personality traits and depression (Qu and Wang, 2024), and also indirectly buffer burnout via teacher resilience (Li, 2023; Xiyun et al., 2022). Qualitative studies indicate Chinese EFL teachers utilize varied strategies to manage negative emotions like exhaustion (Zhao and Wang, 2024) and also actively regulate positive emotionality using both antecedent approaches (e.g., situation selection, cognitive appraisal) and response-focused techniques (e.g., mindfulness, relaxation) (Zhang and Wang, 2024a). Teachers perceive this regulation of positive feelings as important for generating further positivity, improving rapport and classroom climate, and boosting professional success and commitment (Zhang and Wang, 2024a).

Research highlights the significance of emotion regulation (ER) in educational contexts. Teachers regulate emotions for various aims, such as managing their own and students’ feelings for hedonic or instrumental purposes (Taxer and Gross, 2018). Specific strategies are consequential; for example, when responding to student misconduct, teachers relying on cognitive reappraisal experienced fewer negative feelings and engaged in less emotional suppression (Chang and Taxer, 2021), although further investigation into a wider range of ER strategies and their effectiveness is needed. Furthermore, teachers’ perspectives on emotion management techniques and classroom emotional display rules connect to their experiences of burnout (Chang, 2020). Particularly for university EFL lecturers, effective ER is vital for managing the inherent challenges and disappointments within the educational setting (Morris and King, 2018). Research within EFL education suggests that antecedent-focused strategies, especially reappraisal, are effective for enhancing positive emotional expression while reducing negative emotions (Jiang et al., 2016).

Consistent with these findings, EFL teachers’ emotion regulation has been linked to crucial professional outcomes such as teacher self-efficacy and reflection, while showing a negative correlation with burnout. This underscores the importance of exploring ER’s role considering the interplay between perceived school climate, challenging job demands, and teacher burnout specifically among Chinese university EFL educators. A clearer understanding of how specific ER strategies operate in relation to these contextual factors can offer valuable insights for promoting teacher well-being, enhancing classroom management, and ultimately fostering conducive learning environments through evidence-based support and practices.

### 2.6 The purpose of the study

This mixed-methods study investigates the interplay between perceived school climate, challenging job demands, emotion regulation, and teacher burnout among Chinese EFL university instructors. Grounded in the Job Demands-Resources (JD-R) model (Bakker and Demerouti, 2007) and the reviewed literature, the study aims to advance understanding of the mechanisms contributing to teacher burnout in this specific higher education context by examining hypothesized quantitative relationships alongside explorations of lived experiences.

Complementing the quantitative phase, the qualitative component explores the lived experiences of Chinese EFL instructors concerning school climate, job demands, and emotion regulation. Through semi-structured interviews, this phase seeks to contextualize the quantitative results and provide richer, more nuanced insights into factors contributing to or mitigating teacher burnout. This approach aims to foster a more holistic understanding of the phenomenon within this specific setting.

## 3 Methodology

### 3.1 Participants

The initial quantitative phase of this study involved a diverse sample of 478 university EFL instructors. Participants were recruited from various public and private universities across mainland China, representing all three tiers of the national higher education system (as characterized in the Introduction), to ensure a representative cross-section. Eligibility criteria required participants to be currently teaching English, hold valid teaching certification, possess at least 1 year of teaching experience, and be fluent in English.

Demographic analysis of this quantitative sample (N = 478) revealed a balanced gender distribution (54% female, 46% male) and an age range of 31 to 55 years (*M* = 38.71, *SD* = 6.2). Educational qualifications included PhDs (67%) and Master’s degrees (33%). Teaching experience varied: 22% had less than 5 years, 45% had 6 to 10 years, and 33% had over 10 years. Participants taught diverse EFL courses (e.g., language proficiency, literature, communication). Additional demographic data (e.g., socioeconomic status, prior professional development) were collected for potential use as control variables. On average, instructors reported teaching 18 h per week alongside 10 h of administrative duties. From this initial sample, a subsample of 21 instructors was purposively selected for the subsequent qualitative phase (details on selection criteria are provided in Section 3.2).

### 3.2 Procedures

This study employed an explanatory sequential mixed-methods design (QUAN → qual) (e.g., Creswell and Plano Clark, 2018) to investigate the relationships among perceived school climate, challenging job demands, emotion regulation, and teacher burnout among university EFL instructors in mainland China. The research was guided by four primary research questions:

**RQ1:** What is the relationship between perceived school climate and teacher burnout among Chinese EFL university instructors?

**RQ2:** What is the relationship between challenging job demands and teacher burnout among Chinese EFL university instructors?

**RQ3:** Does emotion regulation mediate the relationship between (a) perceived school climate and teacher burnout, and (b) challenging job demands and teacher burnout among Chinese EFL university instructors?

**RQ4:** How do Chinese EFL university instructors perceive and experience the interplay of school climate, job demands, and emotion regulation in relation to their feelings of burnout?

Derived from the quantitative research questions and based on the JD-R model and existing literature, the following hypotheses were tested:

*H1*: Perceived school climate is negatively related to teacher burnout.

*H2*: Challenging job demands are positively related to teacher burnout.

*H3*: Emotion regulation mediates the relationship between perceived school climate and teacher burnout.

*H4*: Emotion regulation mediates the relationship between challenging job demands and teacher burnout.

To address the research questions and test the hypotheses, data were collected using the following procedures, with careful attention to ethical conduct. Data collection for the quantitative phase spanned 20 days and employed a mixed-mode approach for participant convenience. Potential participants received an initial email explaining the study objectives and could choose between completing secure online scales or printed questionnaires distributed by research assistants at their institutions. Ethical considerations were paramount; prior to participation in either mode, individuals were fully informed about the study purpose and procedures and provided written informed consent. The research team strictly adhered to ethical guidelines ensuring voluntary participation, anonymity through de-identification, data confidentiality, and secure data storage.

Subsequently, to enrich and provide nuanced understanding of the quantitative findings, a qualitative phase was conducted. Following preliminary analysis of quantitative data, maximum variation sampling (Patton, 2015) guided the purposive selection of 21 participants from the initial pool (N = 478). This strategy explicitly targeted diversity based on participants’ quantitative scores on burnout, perceived school climate, and emotion regulation profiles (e.g., selecting individuals representing high, medium, and low levels or different patterns) to capture a broad spectrum of experiences related to the core constructs; demographics were reviewed secondarily to ensure a range of contexts. Semi-structured interviews were then conducted individually with these 21 instructors. Using an interview protocol focused on perceptions of climate, demands, and ER strategies, these private interviews lasted approximately 45–60 min each and were audio-recorded with participant consent for subsequent transcription and analysis.

### 3.3 Instruments

To assess the multifaceted nature of teacher burnout among Chinese EFL university instructors, this study employed a comprehensive suite of instruments encompassing both quantitative and qualitative measures.

#### 3.3.1 Challenging Job Demands Scale

The Scale for Challenging Job Demands employed in this study is adapted from Leung et al.’s (2000) questionnaire. To assess faculty members’ experiences with challenging job demands related to teaching, three stressors were selected for evaluation: Teaching Load (TL)—This subscale comprises five items that gauge stressors associated with teaching, such as handling busy teaching schedules and coping with students’ teaching evaluations. New Challenges (NC)—The New Challenges subscale consists of three items aimed at assessing stressors linked to modern educational technology and the need to adapt to new innovations. Research Demands (RD)—The Research Demands subscale includes three items that measure stressors connected to engaging in research work beyond regular teaching hours. Participants rated each item on the Challenge Job Demands Scale using a 6-point scale, ranging from 1 (completely disagree) to 6 (completely agree). Higher scores on the scale indicate a higher perception of stress related to challenging job demands in the teaching role.

#### 3.3.2 Emotion Regulation Scale

The participants’ emotion regulation was evaluated utilizing the Emotion Regulation Scale developed by Gross and John (2003). This survey consists of 10 items specifically designed to assess the participants’ capacity and inclination to manage and control their emotions effectively. The scale includes two fundamental aspects—Expressive Suppression and Cognitive Reappraisal. Each participant was requested to rate the items on a 7-point Likert-type scale, spanning from 1 (completely disagree) to 7 (completely agree). By employing the Emotion Regulation Scale, valuable information was obtained regarding the participants’ approaches to handling their emotional experiences.

#### 3.3.3 School-Level Environment Questionnaire (R-SLEQ)

EFL educators’ perceptions of their school climate were assessed using the School-Level Environment Questionnaire (R-SLEQ) developed by Johnson et al. (2007). This questionnaire comprises 21 items and examines five key dimensions: Collaboration, Student Relations, School Resources, Decision Making, and Instructional Innovation. Participants provided their feedback on a 5-point scale, indicating their level of agreement ranging from 1 (completely disagree) to 5 (completely agree). By employing the R-SLEQ, a thorough insight into the EFL instructors’ viewpoints regarding their school environment was obtained.

#### 3.3.4 Burnout Scale

The Maslach Burnout Scale for educators (MBI-ES), validated and piloted by Maslach et al. (1997), was utilized in this study to evaluate burnout among the participant instructors. The MBI-ES consists of 22 items, encompassing three subscales: Emotional Exhaustion, Depersonalization, and Reduced Personal Accomplishment. Respondents rated each item on a seven-point Likert scale, indicating their frequency of experience, ranging from 0 (never) to 6 (every day). Burnout is measured through lower scores on the Personal Accomplishment subscale and higher scores on the Depersonalization and Emotional Exhaustion subscales.

#### 3.3.5 Semi-structured interviews

To complement the quantitative data and provide a richer understanding of the factors contributing to teacher burnout, semi-structured interviews were conducted with a purposive subsample of 21 participants. An interview guide with broad, open-ended questions was utilized to facilitate in-depth exploration of instructors’ lived experiences related to the study’s key variables. Core guiding questions included:

“Could you describe your typical experiences working within your department and the broader university environment? What aspects stand out to you, both positively and negatively, in terms of support, relationships, autonomy, and decision-making?”

“Thinking about the various expectations placed upon you as an EFL instructor here (related to teaching, research, administration, etc.), how do you perceive these demands, and how do they impact your work experience and overall well-being?”

“Teaching EFL in this context can involve various emotional ups and downs. Can you tell me about your emotional experiences related to your work, and how you typically navigate or manage these feelings, especially during challenging times?”

“Reflecting on these aspects—the work environment, the demands, and the emotional side of teaching—how do you feel they contribute to your overall sense of job satisfaction, stress, or potential burnout?”

The interview guide served as a flexible framework, allowing for follow-up questions and probes based on participant responses to elicit detailed and nuanced information. Conducted in private settings at the participants’ respective universities, the interviews lasted approximately 45–60 min each and were audio-recorded with the participants’ consent for subsequent transcription and analysis.

### 3.4 Data analysis

Prior to the main analyses, the quantitative dataset (N = 478) was screened for missing values. The extent of missing data was minimal (under 3% for any single variable). To handle this missingness under the assumption of data Missing At Random (MAR) without reducing statistical power, the Full Information Maximum Likelihood (FIML) estimation procedure available in AMOS 26.0 was utilized for the CFA and SEM analyses (Schafer and Graham, 2002). FIML uses all available data from each case, providing less biased estimates than traditional deletion methods under MAR. Descriptive statistics, correlation analysis, and reliability indices (using SPSS 28.0) were computed first to gain insights into the sample’s characteristics and examine the bivariate associations between the variables.

To establish the construct validity and model fit for the measurement model, Confirmatory Factor Analysis (CFA) was conducted using AMOS 26.0. CFA allowed for an assessment of the proposed relationships between the latent constructs (school climate, challenging job demands, emotion regulation, and burnout) and their observed indicators (Kline, 2023). Specifically, the measurement model included four latent constructs indicated by their respective items (following the item removals detailed in the Results section): *Perceived School Climate* (indicated by the retained items from the R-SLEQ dimensions), *Challenging Job Demands* (indicated by the retained items from its three subscales: Teaching Load, New Challenges, Research Demands), *Emotion Regulation* (indicated by items from its two subscales: Cognitive Reappraisal, Expressive Suppression), and *Teacher Burnout* (indicated by the retained items from its three subscales: Emotional Exhaustion, Depersonalization, and reverse-scored Personal Accomplishment). To test the hypothesized model and examine the mediating role of emotion regulation, Structural Equation Modeling (SEM) was employed. Furthermore, mediation analysis was utilized to investigate whether emotion regulation acts as a mediator between perceived school climate and challenging job demands (independent variables) and burnout (dependent variable) among EFL teachers. Bootstrapping, with 50,000 resampled datasets and bias-corrected 95% confidence intervals, was employed to determine the significance of the mediating role of emotion regulation, as it is particularly advantageous when dealing with non-normal data or relatively small sample sizes (Cheung and Lau, 2008). Model fit was evaluated using well-established fit indices, including the ratio of χ2 goodness of fit to the degree of freedom (df), the Comparative Fit Index (CFI), the Tucker-Lewis Index (TLI), the Root-Mean-Square Error of Approximation (RMSEA), and the Standardized Root-Mean-Square Residual (SRMR) (Vandenberg and Lance, 2000). Conventional cutoffs, drawing on recommendations from sources such as Hu and Bentler (1999) and Kline (2023), were used to evaluate model fit (e.g., *χ^2^*/*df* < 3, *CFI*/*TLI* ≥ 0.90, *RMSEA* ≤ 0.08, *SRMR* ≤ 0.08).

The qualitative data gleaned from the semi-structured interviews were meticulously transcribed verbatim in Chinese (approx. 350,000 characters). These transcripts were then carefully translated into English by two independent bilingual researchers; discrepancies were resolved through discussion to ensure translation fidelity, yielding a final corpus of approximately 240,000 English words for analysis. The translated data were subsequently analyzed using thematic analysis following the procedures outlined by Braun and Clarke (2006), a method focused on identifying, analyzing, and reporting patterns (themes) within textual data. The analysis was conducted iteratively by the lead author and a trained graduate research assistant using NVivo (Version 12) software to manage the data; coding consistency was ensured through independent coding of a subset of transcripts followed by discussion to resolve discrepancies and refine the codebook, with remaining transcripts coded by one researcher and checked by the other.

Positionality and reflexivity were central to the qualitative analysis process. The lead author, with prior experience as an EFL instructor in Chinese higher education, possessed an insider perspective facilitating rapport and contextual understanding, while also necessitating conscious bracketing of assumptions. The research assistant provided a complementary external perspective grounded in applied linguistics but without prior experience in the specific institutions. To mitigate potential biases and enhance interpretative rigor and trustworthiness, both researchers maintained reflexive journals and engaged in regular peer debriefing sessions to critically examine emergent codes and themes, challenge assumptions, and consider alternative interpretations.

Following Braun and Clarke's (2006) six-phase framework, the analysis involved systematic steps: initial familiarization through repeated reading; generating 482 initial codes across the dataset capturing relevant meanings; collating codes into potential themes; iteratively reviewing and refining themes against the data and research questions; defining and naming final themes; and compiling the final report with illustrative quotes to ensure analytic depth and authenticity.

## 4 Results

### 4.1 Quantitative results

To establish the construct validity and robustness of the measurement model, Confirmatory Factor Analysis (CFA) was conducted using AMOS 26.0. CFA allowed for examining the proposed relationships between the latent constructs (school climate, challenging job demands, emotion regulation, and burnout) and their observed indicators, and assessing the model fit to the data. The initial measurement model included indicators for the four latent constructs, with each construct represented by multiple observed indicators derived from their respective scales. The initial four-factor measurement model yielded the following fit indices: *χ^2^*(*df*) = 286 (144), *p* < 0.001, *CFI* = 0.911, *TLI* = 0.893, *RMSEA* = 0.086 (90% CI [0.073, 0.099]), *SRMR* = 0.071. While some indices approached acceptable levels, overall model fit improvement was warranted to achieve good fit according to recommended criteria (e.g., Hu and Bentler, 1999; Kline, 2023).

Model modification proceeded iteratively, adhering to recommended practices (Bollen, 1989; Kline, 2023). At each step, modification indices (MIs) and standardized factor loadings were carefully examined to identify sources of misfit. Decisions to modify the model, primarily involving the removal of problematic items, were guided by large MIs suggesting significant cross-loadings or correlated error terms, and by weak factor loadings. Aiming for strong indicator reliability and clear construct representation, and consistent with recommendations for retaining items that strongly represent the intended factor (Hair et al., 2010), items with standardized factor loadings below a threshold of 0.50 were considered candidates for removal, particularly if also flagged by MIs or deemed theoretically less central to the construct’s core definition. Modifications were implemented sequentially, one item at a time, starting with the item exhibiting the most substantial evidence of misfit (e.g., lowest loading combined with high MIs). Model fit was re-assessed after each modification using the standard fit indices, and the statistical significance of the improvement in fit was evaluated using the chi-square difference test (Δ*χ^2^*) against the change in degrees of freedom (Δ*df*), with *p* < 0.05 indicating a significant improvement (Bollen, 1989).

This iterative process resulted in the sequential removal of five items. Table 1 details the fit statistics at each step. Briefly, the item removals proceeded as follows: (1) Removal of Burnout item “I feel frustrated by my teaching responsibilities” (*λ* = 0.48 in initial model), which significantly improved fit [Δ*χ^2^*(1) = 21.0, *p* < 0.001]. (2) Removal of School Climate item “There is a lack of respect among staff members” (λ = 0.46 in Model 1), leading to further significant improvement [Δ*χ^2^*(1) = 17.0, *p* < 0.001]. (3) Removal of School Climate item “Teachers in this school are often indifferent to each other’s problems” (λ = 0.42 in Model 2), significantly improving fit [Δ*χ^2^*(1) = 15.0, *p* < 0.001]. (4) Removal of Job Demands item “The administrative tasks I am required to complete are overwhelming” (λ = 0.47 in Model 3), resulting in significant fit improvement [Δ*χ^2^*(1) = 13.0, *p* < 0.001]. (5) Removal of Burnout item “I feel emotionally drained at the end of each teaching day” (λ = 0.45 in Model 4), which yielded the final significant improvement [Δ*χ^2^*(1) = 8.0, *p* < 0.01]. No further modifications were deemed necessary as the final model achieved good fit and remaining items exhibited acceptable loadings and no major sources of misfit were indicated by MIs.

**Table 1:** Summary of CFA measurement model fit statistics across modification steps.

Model	*χ^2^*	*df*	*p*	Δ*χ^2^*	Δ*df*	Δ*χ^2^ p*	*CFI*	*TLI*	*RMSEA* [90% CI]	*SRMR*	Notes on Modification
Model 0: Initial	286.0	144	<0.001	-	-	-	0.911	0.893	0.086 [0.073, 0.099]	0.071	Baseline 4-factor model
Model 1: Remove BU4	265.0	143	<0.001	21.0	1	<0.001	0.925	0.905	0.080 [0.068, 0.092]	0.065	Removed “frustrated.”
Model 2: Remove SC12	248.0	142	<0.001	17.0	1	<0.001	0.935	0.915	0.075 [0.062, 0.087]	0.060	Removed “lack of respect.”
Model 3: Remove SC8	233.0	141	<0.001	15.0	1	<0.001	0.942	0.924	0.070 [0.057, 0.082]	0.056	Removed “indifferent.”
Model 4: Remove JD5	220.0	140	0.002	13.0	1	<0.001	0.948	0.933	0.065 [0.052, 0.077]	0.053	Removed “admin tasks.”
Model 5: Final Revised	212.0	139	0.005	8.0	1	<0.01	0.953	0.941	0.061 [0.048, 0.073]	0.051	Removed “drained.”

Model modifications were made sequentially based on examination of standardized factor loadings (< 0.50) and modification indices. Δ*χ*^2^ test compares fit against the preceding model. BU, Burnout; SC, School Climate, JD, Job Demands. *p*-values for Δ*χ*^2^ based on *χ*^2^ distribution with Δ*df*. Final model (Model 5) used for subsequent analyses.

After implementing these sequential, theoretically, and empirically justified modifications, the final revised measurement model was evaluated. As detailed in Table 1 (Model 5), the revised model demonstrated good fit to the data, indicating enhanced model fit and construct validity. The fit indices for the final revised model were: *χ^2^*(*df*) = 212 (139), *p* = 0.005, *CFI* = 0.953, *TLI* = 0.941, *RMSEA* = 0.061 (90% CI [0.048, 0.073]), *SRMR* = 0.051. Comparison of the fit indices across the iterations confirms the improvement achieved through this rigorous modification process. Based on the final model’s good fit and enhanced construct validity, it was considered appropriate for subsequent data analyses, including SEM to examine the hypothesized relationships between the constructs.

Next, convergent and discriminant validity were assessed (see Table 2). The Average Variance Extracted (*AVE*) and Composite Reliability (*CR*) (Fornell and Larcker, 1981) were computed to evaluate the convergent validity and internal consistency reliability of the latent constructs, respectively. The *AVE* represents the amount of variance captured by each construct relative to measurement error, while *CR* assesses the shared variance among the indicators of a construct, often considered a more suitable reliability measure than Cronbach’s alpha within an SEM framework (Hair et al., 2010).

**Table 2:** Convergent and divergent validity.

Variables	AVE (AVE Square Root)	CR	1	2	3	4
1. School Climate	0.60 (0.77)	0.85	(0.77)			
2. Job Demands	0.55 (0.74)	0.80	−0.32*	(0.74)		
3. Emotion Regulation	0.65 (0.81)	0.89	0.21**	−0.29**	(0.81)	
4. Burnout	0.58 (0.76)	0.82	−0.44***	0.33***	−0.38***	(0.76)

AVE, Average Variance Extracted; CR, Composite Reliability. Square roots of AVE are shown in bold on the diagonal. Correlations are shown below the diagonal. *p* < 0.05, *p < 0.01, **p < 0.001.

The results of the convergent and discriminant validity assessment provide evidence for the soundness of the measurement model. The *AVE* values exceeding the recommended threshold of 0.50 for all constructs (Fornell and Larcker, 1981; Hair et al., 2010) demonstrate that each latent variable accounts for more than half of its variance, indicating adequate convergent validity. Additionally, the *CR* values exceeding the recommended threshold of 0.70 for all constructs (Fornell and Larcker, 1981; Hair et al., 2010) indicate satisfactory internal consistency reliability. Discriminant validity was assessed using the Fornell-Larcker criterion (Fornell and Larcker, 1981). As presented in Table 2, the square root of the *AVE* for each construct (values shown in bold on the diagonal) is greater than its correlation coefficients with all other latent constructs in the model (off-diagonal values). This confirms that each construct shares more variance with its own indicators than with other constructs, establishing discriminant validity. The findings support the overall validity of the measurement model and provide assurance that the measurement instruments accurately capture distinct underlying constructs among EFL teachers.

Descriptive statistics and reliability indices for the variables are presented in Table 3. Mean scores for school climate, job demands, emotion regulation, and burnout were 3.67 (SD = 0.82), 4.12 (SD = 0.93), 5.24 (SD = 1.10), and 2.95 (SD = 0.78), respectively. Skewness and kurtosis values indicated that the data generally approximated normality. Internal consistency reliability for the observed variable scales was also assessed using Cronbach’s alpha (*α*). The coefficients revealed acceptable to excellent reliability, typically indicated by α ≥ 0.70 (e.g., Nunnally and Bernstein, 1994), for all scales (school climate: α = 0.85; job demands: α = 0.79; emotion regulation: α = 0.91; burnout: α = 0.88).

**Table 3:** Descriptive statistics and reliability indices.

Variables	Mean	SD	Skewness	Kurtosis	Cronbach’s Alpha
School Climate	3.67	0.82	−0.24	1.12	0.85
Job Demands	4.12	0.93	0.08	1.01	0.79
Emotion Regulation	5.24	1.10	−0.50	1.56	0.91
Burnout	2.95	0.78	0.40	0.92	0.88

Correlations between the constructs are shown in Table 4. School climate was negatively correlated with job demands (r = −0.32, *p* < 0.05) and positively correlated with emotion regulation (r = 0.21, *p* < 0.01). School climate was also negatively correlated with burnout (r = −0.44, *p* < 0.001), while job demands were positively correlated with burnout (r = 0.33, *p* < 0.001). Emotion regulation was negatively correlated with both job demands (r = −0.29, *p* < 0.01) and burnout (r = −0.38, *p* < 0.001). These correlations provide initial support for the hypothesized relationships.

**Table 4:** Correlations among the constructs.

Variables	1	2	3	4
1. School Climate	1.00			
2. Job Demands	−0.32*	1.00		
3. Emotion Regulation	0.21**	−0.29**	1.00	
4. Burnout	−0.44***	0.33***	−0.38***	1.00

*p < 0.05, **p < 0.01, ***p < 0.001.

**Figure 1:**
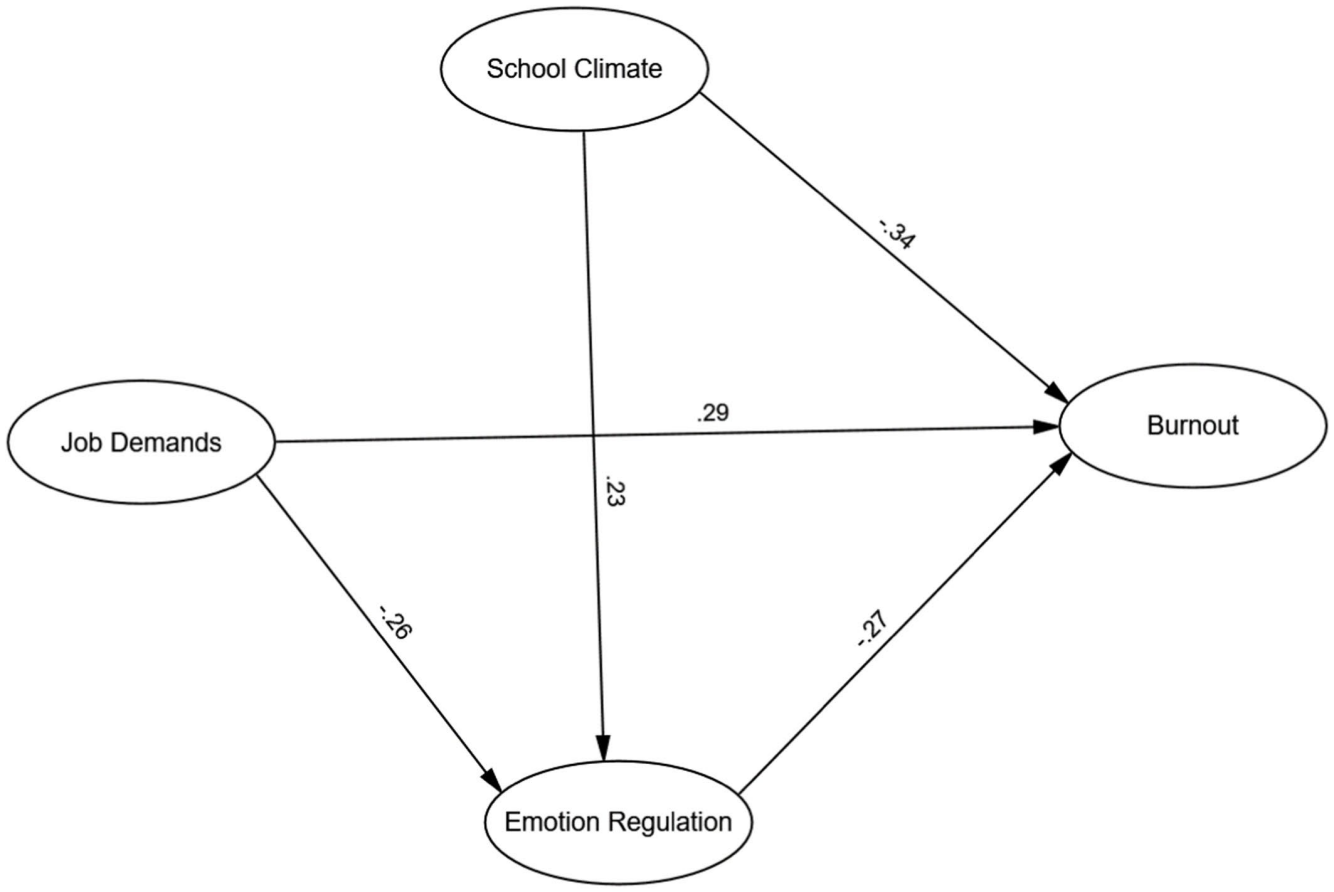
The mediation model.

The hypothesized model was evaluated using SEM which is a powerful statistical technique, allowing for the simultaneous examination of multiple relationships between latent constructs and their observed indicators. The SEM analysis yielded highly favorable results, demonstrating an exceptional fit of the model to the data. The goodness-of-fit indices supported the adequacy of the model, as evidenced by the following statistics: χ^2^ (4) = 7.89, *p* = 0.096, CFI = 0.98, TLI = 0.96, and RMSEA = 0.04 with a 90% confidence interval (CI) ranging from 0.00 to 0.08. The path coefficients are indicated in Figure 1. Moreover, the model accounted for a substantial proportion of the variance in teacher burnout, explaining 36.3% of the variance in this critical outcome variable. This finding underscores the significance of the proposed relationships in understanding teacher burnout among Chinese EFL instructors.

Mediation analysis examined whether emotion regulation mediated the relationships between perceived school climate, challenging job demands (independent variables), and burnout (dependent variable) among EFL teachers. Bootstrapping with 50,000 resampled datasets and bias-corrected 95% confidence intervals was employed to assess the significance of the mediation (indirect) effects, as this approach ensures robust and reliable estimates even with non-normal data or smaller sample sizes (Cheung and Lau, 2008; Efron and Tibshirani, 1993).

The analysis revealed significant direct and indirect effects (see Table 5). Perceived school climate was negatively associated with teacher burnout (β = −0.34, *p* < 0.001), indicating that a positive school climate directly reduces burnout. Challenging job demands showed a positive association with burnout (β = 0.29, *p* < 0.001), while emotion regulation was negatively related to burnout (β = −0.27, *p* < 0.001), suggesting that effective emotion regulation lowers burnout levels.

**Table 5:** Direct, indirect, and total effects in mediation analysis.

Path	Coefficient (β)	Bootstrapped 95% CI	*p*-value	Effect type
School Climate → burnout	−0.34	[−0.41, −0.28]	<0.001	Direct
Job Demands → burnout	0.29	[0.24, 0.35]	<0.001	Direct
Emotion Regulation → burnout	−0.27	[−0.33, −0.22]	<0.001	Direct
School Climate → burnout	−0.06	[−0.10, −0.02]	0.005	Indirect
Job Demands → burnout	0.07	[0.03, 0.12]	<0.001	Indirect
School Climate → burnout (Total)	−0.40	[−0.46, −0.34]	<0.001	Total
Job Demands → burnout (Total)	0.36	[0.30, 0.42]	<0.001	Total

CI, Confidence interval.

For indirect effects, emotion regulation significantly mediated the relationship between school climate and burnout (indirect effect β = −0.06, 95% CI [−0.10, −0.02], *p* = 0.005), as well as between job demands and burnout (indirect effect β = 0.07, 95% CI [0.03, 0.12], *p* < 0.001). Crucially, the simultaneous statistical significance of both the direct effects (School Climate → Burnout; Job Demands → Burnout) and their respective indirect effects through emotion regulation indicates support for partial mediation for both proposed pathways (Baron and Kenny, 1986; Zhao et al., 2010). This pattern suggests that emotion regulation is an important mechanism that explains part of how school climate and job demands influence teacher burnout, but these work characteristics also continue to exert a significant direct influence on burnout independent of the mediating role of emotion regulation. These findings underscore emotion regulation’s role as an important, but not exclusive, pathway linking school climate and job demands to burnout experiences among EFL instructors. Total effects, combining direct and indirect influences, highlight the overall substantive impact of both school climate and job demands on burnout levels among EFL teachers. Table 5 summarizes these mediation effects, illustrating the significant direct and indirect pathways.

Ultimately, to ensure that the mediation model operates equivalently across gender, measurement invariance tests were conducted. Establishing measurement invariance is crucial to confirm that the constructs (school climate, job demands, emotion regulation, and burnout) are measured similarly for both male and female instructors, allowing for meaningful comparisons.

Configural Invariance: The first step involved testing configural invariance, which assesses whether the same factor structure is applicable across groups. The configural model, which allows for different parameter estimates across groups, demonstrated an acceptable fit across both male and female instructors (CFI = 0.921, TLI = 0.904, RMSEA = 0.065). This indicates that the basic structure of the constructs is similar for both genders. Next, metric invariance was tested by constraining the factor loadings to be equal across genders. This step examines whether the relationships between items and their respective constructs are equivalent. The model fit indices for metric invariance were: χ^2^(df) = 312 (152), CFI = 0.915, TLI = 0.898, RMSEA = 0.068. The change in fit indices (ΔCFI < 0.01, ΔRMSEA < 0.015) supported metric invariance, indicating that the strength of the relationships between items and constructs is consistent across male and female instructors.

To further test scalar invariance, item intercepts were constrained to be equal across genders. This step ensures that the same constructs have the same meaning and can be compared quantitatively between groups. The model fit indices for scalar invariance were: χ^2^(df) = 334 (160), CFI = 0.908, TLI = 0.890, RMSEA = 0.070. The change in fit indices (ΔCFI < 0.01, ΔRMSEA < 0.015) indicated that scalar invariance was supported, suggesting that the latent constructs have the same meaning across genders. Finally, strict invariance was tested by constraining both item residuals and intercepts to be equal across genders. The model fit indices for strict invariance were: χ^2^(df) = 356 (168), CFI = 0.902, TLI = 0.884, RMSEA = 0.073. The changes in fit indices (ΔCFI < 0.01, ΔRMSEA < 0.015) confirmed that strict invariance was supported, indicating that the measurement model operates equivalently across male and female instructors. These results confirm that the measurement model works equivalently for both male and female instructors, allowing for valid and meaningful comparisons between these groups in subsequent analyses.

### 4.2 Qualitative results

The qualitative analysis of the semi-structured interviews, guided by Braun and Clarke's (2006) six-phase thematic analysis framework, provided rich insights into the lived experiences of Chinese EFL university instructors. The analysis revealed a complex interplay of factors contributing to their perceptions of school climate, job demands, emotion regulation, and burnout. Three overarching themes emerged from the data: (1) The Supportive yet Stifling School Climate, (2) The Weight of Unrealistic Expectations, and (3) Navigating the Emotional Landscape. These themes are elaborated below, with illustrative quotes from participants to provide context and authenticity.

#### 4.2.1 The supportive yet stifling school climate

This theme encapsulates the paradoxical nature of the school climate experienced by the EFL instructors. While they expressed appreciation for the supportive and collaborative relationships with their colleagues, they simultaneously felt constrained by a lack of autonomy and a rigid hierarchical structure within their institutions.

##### 4.2.1.1 Collegiality and support

Many participants spoke positively about the sense of community and camaraderie within their departments. They valued the opportunity to connect with colleagues who shared similar experiences and could offer support and guidance. This sense of belonging was evident in statements like, “We have a really good team spirit in our department. We often help each other out with teaching materials, share ideas, and provide feedback. It’s a very supportive environment” (Participant 12). This collaborative spirit fostered a sense of shared responsibility and mutual support, which seemed to buffer against some of the stressors associated with their demanding roles. Another participant expressed similar sentiments, stating, “I feel fortunate to work with such a great group of colleagues. We have a strong sense of community, and we are always there to support each other, both personally and professionally” (Participant 5). These comments suggest that positive relationships with colleagues can be a significant source of resilience and job satisfaction for EFL instructors.

##### 4.2.1.2 Lack of autonomy and flexibility

Despite the positive aspects of collegial support, a contrasting narrative emerged, highlighting a perceived lack of autonomy and flexibility in their teaching roles. Participants felt constrained by standardized curricula, which limited their ability to innovate and tailor their teaching approaches to the specific needs of their students. “There’s not much room for creativity in our teaching,” one participant lamented. “We have to follow the prescribed textbooks and syllabus very closely. It can feel quite stifling at times” (Participant 8). This perceived lack of agency was a source of frustration for many participants, who felt their pedagogical expertise and creativity were being underutilized. Another participant expressed a similar concern, stating, “I wish we had more freedom to experiment with different teaching approaches and tailor our lessons to the specific needs of our students. The rigid curriculum can make it difficult to be truly innovative” (Participant 16). This suggests that a lack of autonomy can lead to feelings of dissatisfaction and may even stifle professional growth.

##### 4.2.1.3 Hierarchical structure and undervaluation

Adding to this sense of constraint, participants also perceived a rigid hierarchical structure within the university system, which contributed to feelings of being undervalued and having limited influence on decision-making processes. “I feel like my expertise is not fully recognized,” shared one participant. “We are often told what to do and how to do it, even though we have years of experience and advanced degrees” (Participant 17). This perceived lack of respect for their professional judgment was echoed by another participant who expressed frustration with top-down decision-making: “It can be frustrating when decisions are made at the top without any consultation with the instructors who are actually doing the teaching” (Participant 20). These findings indicate that a rigid hierarchy can create a sense of powerlessness and contribute to feelings of being undervalued, potentially leading to disengagement and burnout.

#### 4.2.2 The weight of unrealistic expectations

This theme vividly illustrates the pervasive sense of pressure experienced by EFL instructors due to the heavy workload and unrealistic expectations placed upon them by their institutions. The competing demands of teaching, research, and administrative duties often left them feeling overwhelmed, exhausted, and struggling to maintain a healthy work-life balance. This constant pressure created a sense of being caught in a perpetual struggle to meet the demands of their multifaceted roles.

##### 4.2.2.1 Heavy workload

Participants consistently described a demanding work schedule that often extended beyond regular working hours, leaving them with little time for personal pursuits or rest. They spoke of juggling a high volume of teaching hours, extensive grading responsibilities, frequent meetings, and a seemingly endless stream of administrative tasks. One participant vividly captured this experience: “The workload is just relentless. We have so many classes to teach, papers to grade, and meetings to attend. On top of that, we are expected to publish regularly in top-tier journals. It’s just too much” (Participant 3). This feeling of being overburdened was echoed by another participant who spoke of the encroachment of work into their personal time: “I often find myself working late into the night and on weekends just to keep up with everything. It’s exhausting and leaves me with little time for myself or my family” (Participant 11). These accounts paint a picture of constant pressure and a struggle to maintain boundaries between work and personal life, a situation that can easily lead to chronic stress and burnout.

##### 4.2.2.2 Pressure to prioritize research

Adding to this already heavy workload, many participants felt a pressure to prioritize research over teaching, creating a sense of conflict and dissatisfaction. They felt that their institutions placed a higher value on research output than on teaching performance, even though many derived greater personal satisfaction from teaching and interacting with students. “I love teaching, but I feel like I have to focus on research if I want to get promoted. It’s a constant struggle to balance the two” (Participant 9). This pressure to prioritize research, often at the expense of teaching, can lead to feelings of guilt and inadequacy for those who are passionate about their role as educators. Another participant expressed a similar concern, stating, “I sometimes feel like my teaching is an afterthought. The university puts so much emphasis on research publications, and it can feel like my teaching contributions are not truly appreciated” (Participant 14). This perceived imbalance between the value placed on research versus teaching can create a sense of dissonance and contribute to feelings of being undervalued and demoralized.

##### 4.2.2.3 Emphasis on student evaluations

Further contributing to the pressure cooker environment, participants expressed concerns about the increasing emphasis on student evaluations, which they felt often prioritized student satisfaction over pedagogical quality. They felt pressured to cater to students’ preferences, even if it meant compromising their teaching approaches or lowering academic standards. “We are constantly being evaluated by our students, and it feels like we have to cater to their every whim to get good ratings. It can be very demoralizing” (Participant 15). This constant scrutiny and pressure to please students can create anxiety and undermine instructors’ confidence in their pedagogical decisions. Another participant expressed concern about the potential impact on academic standards: “I worry that the focus on student evaluations is leading to a decline in academic rigor. We are encouraged to make our courses easier and more entertaining, even if it means sacrificing depth and critical thinking” (Participant 18). These findings suggest that an overemphasis on student evaluations can create a conflict between meeting student expectations and maintaining high academic standards, potentially leading to a sense of compromise and dissatisfaction among instructors.

#### 4.2.3 Navigating the emotional landscape

This theme delves into the emotional challenges inherent in the teaching profession, highlighting the importance of emotion regulation for EFL instructors. Participants demonstrated an awareness of these challenges and discussed various strategies they employed to manage their emotions, as well as the difficulties they encountered in doing so.

##### 4.2.3.1 Emotion regulation strategies

Participants recognized the importance of emotion regulation in their work and described a range of strategies they utilized to navigate the emotional ups and downs of the teaching profession. These strategies included cognitive reframing, seeking support from colleagues, practicing mindfulness, and prioritizing self-care. One participant shared, “When I’m feeling stressed, I try to reframe the situation and focus on the positive aspects of my job. I also find it helpful to talk to my colleagues and get their support” (Participant 6). This proactive approach to managing stress highlights the importance of both cognitive and social resources in emotion regulation. Another participant emphasized the role of mindfulness and self-care: “I practice mindfulness meditation regularly, which helps me to stay calm and focused, even when things get hectic. I also make sure to prioritize my physical and mental health by exercising, getting enough sleep, and spending time with loved ones” (Participant 10). This holistic approach to well-being recognizes the interconnectedness of physical, mental, and emotional health in promoting resilience and preventing burnout.

##### 4.2.3.2 Challenges in emotion regulation

While participants demonstrated a commitment to emotion regulation, some acknowledged the difficulties they faced in managing their emotions effectively, particularly when confronted with challenging student behaviors or negative feedback. One participant candidly shared, “Sometimes I just feel overwhelmed by the negativity. It can be really draining to deal with difficult students or critical comments from colleagues” (Participant 19). This highlights the emotional toll that challenging interpersonal interactions can take on instructors, even those who are generally skilled at emotion regulation. Another participant acknowledged the ongoing process of developing emotion regulation skills: “I’m still learning how to regulate my emotions effectively. I sometimes struggle with feelings of frustration and self-doubt, especially when I feel like I’m not meeting everyone’s expectations” (Participant 2). This emphasizes that emotion regulation is a continuous journey that requires ongoing effort and self-reflection.

##### 4.2.3.3 Emotional intelligence

Participants also emphasized the importance of emotional intelligence in navigating the complex interpersonal dynamics of the university environment. They recognized the need to be attuned to their own emotions and the emotions of others, and to communicate effectively in emotionally charged situations. “You have to be able to read people and understand their emotions. It’s essential for building positive relationships with students and colleagues” (Participant 21). This highlights the importance of empathy and social awareness in creating a positive and supportive learning environment. Another participant connected emotional intelligence to effective teaching: “Emotional intelligence is crucial for effective teaching. You need to be able to create a positive and supportive learning environment, and that requires a high degree of emotional awareness and empathy” (Participant 7). This underscores the role of emotional intelligence in fostering positive teacher-student relationships and creating a classroom climate conducive to learning.

Taken together, the qualitative findings complemented the quantitative results, providing a richer understanding of the relationships between perceived school climate, job demands, emotion regulation, and teacher burnout. For instance, the qualitative data illustrated how a lack of autonomy and a rigid hierarchy could lead to feelings of frustration and burnout, supporting the quantitative finding of a negative relationship between school climate and burnout. Similarly, the qualitative data showed how pressure to prioritize research and an emphasis on student evaluations could contribute to emotional exhaustion, aligning with the positive relationship found between job demands and burnout. Furthermore, the qualitative data highlighted the importance of emotion regulation strategies in mitigating stress, supporting the finding that emotion regulation mediates the relationship between school climate, job demands, and burnout.

## 5 Discussion

The aim of the present mixed-methods study was to investigate the relationship between perceived school climate, job demands, and teacher burnout through the mediating role of teacher emotion regulation among Chinese university EFL teachers. The findings shed valuable light on the factors influencing teacher well-being and emphasize the mediating role of emotion regulation in this context.

It was found that emotion regulation directly and negatively predicts teacher burnout. This study supports a growing body of research establishing a substantial relationship between emotional regulation and teacher burnout (e.g., Atmaca et al., 2020; Chang, 2020; Ma, 2023; Mulyani et al., 2021; Wang et al., 2024). Teachers with higher emotion regulation abilities exhibit lower levels of burnout, reinforcing the importance of emotional regulation in buffering the negative consequences of job-related stressors (Chang and Taxer, 2021; Li, 2023; Mérida-López and Extremera, 2017; Qu and Wang, 2024). Qualitative findings underscore this point by highlighting how teachers employ strategies such as cognitive reframing, mindfulness, and collegial support to navigate emotional challenges effectively. For example, participants emphasized the value of talking with colleagues to reframe stressful situations and reduce the emotional toll of teaching, which aligns with quantitative evidence on the protective role of emotion regulation.

Instructors who are better able to control and manage their feelings are better able to handle challenging circumstances and are less likely to suffer from emotional exhaustion. Consistent with findings linking positive affect and effective regulation to better coping (e.g., Chan, 2006; Zhang and Wang, 2024a), enhancing instructors’ positive feelings alongside adaptive regulation strategies may aid them in overcoming fatigue, fostering empathy, and lowering depersonalization. Instructors’ ability to regulate their emotions effectively could lead to greater personal accomplishment and work engagement (Ma, 2023). Emotional exhaustion, a core component of burnout (Maslach, 2003), manifests in various ways, and the qualitative findings offer a nuanced understanding, with participants describing the draining nature of dealing with demanding students or critical feedback. However, the interviews also revealed how proactive strategies like mindfulness and self-care can mitigate these effects (Liu and Du, 2024).

Moreover, confirming a central tenet of the JD-R model, challenging job demands were directly associated with teacher burnout. Workplace demands require sustained effort and can impair well-being by depleting physical and psychological resources, potentially leading to burnout symptoms (Bakker and Demerouti, 2017). Teachers facing high job demands in this study, consistent with much previous research, were indeed more susceptible to experiencing emotional exhaustion and reduced personal accomplishment (e.g., Alarcon, 2011; Bottiani et al., 2019). As job demands increase, teachers may find it challenging to effectively manage their resources and cope with stressors, leading to heightened burnout levels (Galanakis and Tsitouri, 2022; Han et al., 2020; Hu et al., 2023). The qualitative findings provide concrete examples, with participants vividly describing how heavy teaching loads, research pressures, extensive grading, and administrative duties encroached on personal time, leading to feelings of being overwhelmed and exhausted.

In line with this finding, research applying the JD-R model frequently observes positive associations between workload or other demands and burnout indicators (e.g., Galanakis and Tsitouri, 2022; Schaufeli and Bakker, 2004). Furthermore, specific demands prominent in the Chinese academic setting, such as high research expectations conflicting with teaching roles or pressures related to work-life balance, are recognized contributors to burnout (Han et al., 2020; Hu et al., 2023; Yin et al., 2018). The qualitative theme of “The Weight of Unrealistic Expectations” complements these findings by illustrating how the prioritization of research over teaching exacerbated feelings of inadequacy and discontent among instructors, compounding their emotional exhaustion and depersonalization.

In addition, the results revealed a significant direct and negative association between perceived school climate and teacher burnout, indicating that positive climates relate to lower burnout. This finding aligns with research consistently showing the beneficial impact of school climate on teacher well-being and job satisfaction across educational levels (e.g., Aldridge and McChesney, 2018; Malinen and Savolainen, 2016; Yang et al., 2022; Zeng et al., 2024). A positive and supportive school climate fosters a sense of belonging and community, which can buffer against stress and emotional exhaustion (Ibrahim and El Zaatari, 2020; Yin et al., 2018). Qualitative data added depth to this understanding by revealing the duality of a supportive yet stifling school climate. While participants valued collegiality, many felt constrained by hierarchical decision-making and lack of autonomy, undermining potential climate benefits.

Furthermore, a positive school climate can promote effective communication and collaboration among teachers and school administrators (Gray et al., 2017). When teachers feel that they are part of a cohesive team working towards shared goals, they may experience reduced feelings of isolation and emotional strain, contributing to lower levels of burnout (Dutta and Sahney, 2022). However, the qualitative findings also highlighted the emotional toll of feeling undervalued within rigid hierarchical structures. Participants described how top-down decision-making and a lack of recognition for their expertise led to feelings of frustration and disengagement, which align with the quantitative findings on the negative relationship between school climate and burnout.

Finally, the mediation analysis further illuminated the crucial role of emotion regulation as a mechanism linking work conditions to burnout among these EFL instructors. The quantitative findings supported partial mediation, revealing two significant indirect pathways through which emotion regulation operates: First, a positive school climate appeared to foster lower burnout partly *through* enhanced emotion regulation. Instructors perceiving a more positive climate reported better emotion regulation skills, which in turn predicted lower levels of burnout (Indirect β = −0.06). This suggests that supportive, collaborative environments, functioning as job resources, may bolster instructors’ capacity to manage emotions effectively, perhaps by increasing positive affect or providing psychological safety (Chang et al., 2022; Zeng et al., 2024). This protective mechanism resonates with the qualitative data, where participants described leveraging collegial support within positive climates to reframe difficulties and mitigate negative feelings.

Second, challenging job demands seemed to contribute to higher burnout partly *through* hindered emotion regulation. Higher reported job demands were associated with lower levels of emotion regulation, which subsequently predicted increased burnout (Indirect β = 0.07). This pathway aligns with the JD-R model’s health impairment process; excessive demands likely deplete not only cognitive and physical energy but also the personal resources needed for effective emotion regulation (Bakker and Demerouti, 2017). Such depletion was evident in qualitative accounts of instructors feeling overwhelmed, emotionally drained, and struggling with frustration under relentless workloads and pressures, indicating difficulties in emotional management when demands were high (Galanakis and Tsitouri, 2022; Hu et al., 2023). Proactive strategies like mindfulness and self-care, mentioned by some participants, likely represent attempts to counteract this erosion of regulatory resources (Liu and Du, 2024).

Taken together, these mediation results underscore that emotion regulation capability interacts dynamically with the work environment. A positive climate appears to facilitate effective regulation, whereas high demands seem to impede it, both processes significantly influencing teacher burnout (Brackett et al., 2010; Mulyani et al., 2021; Wang et al., 2024). The qualitative emphasis on emotional intelligence—requiring empathy and social awareness to navigate complex interpersonal dynamics within the university—further highlights the practical importance of these regulatory skills in this context.

Overall, the integration of qualitative findings provides a richer and more nuanced understanding of the relationships between school climate, job demands, emotion regulation, and teacher burnout. The qualitative themes offer real-life examples and emotional narratives that illuminate the mechanisms identified quantitatively, reinforcing the critical and interactive role of emotion regulation in shaping the impact of school climate and job demands on teacher well-being.

## 6 Conclusion and implications

This mixed-methods study investigated the relationships between perceived school climate, job demands, emotion regulation, and teacher burnout among Chinese EFL university instructors. Results showed that a positive school climate reduces burnout, while challenging job demands increase it. Emotion regulation was found to partially mediate these relationships and promote resilience. Qualitative findings provided nuanced insights into teachers’ experiences, highlighting the duality of school climate (supportive yet stifling), the emotional toll of heavy workloads and competing expectations, and the strategies used to navigate emotional challenges. These findings underscore the importance of fostering both supportive environments and emotional resilience in educators.

The implications of this research are significant for educational institutions, policymakers, and teacher support programs. This study underscores the necessity of cultivating a positive and supportive school climate that promotes respect, collaboration, and professional autonomy. Qualitative data emphasized that a lack of autonomy and hierarchical decision-making undermines teachers’ sense of agency, highlighting the need for systemic changes initiated by institutional leadership that empower educators. To address these challenges, institutions should prioritize evaluating and restructuring decision-making processes to ensure meaningful faculty involvement and foster a culture where teacher expertise and pedagogical judgment are genuinely valued. Training and organizational development efforts focused on enhancing supportive leadership competencies, promoting participatory management styles, and ensuring transparent communication channels should be directed towards administrators and academic leaders. While professional development opportunities for instructors focusing on enhancing collegial collaboration and communication skills remain valuable, these should be implemented judiciously, ensuring they do not exacerbate existing workload pressures. Critically, such initiatives must complement, rather than substitute for, genuine top-down commitment to improving structural issues related to autonomy and hierarchy.

In addition to improving school climate, mentorship programs where experienced teachers support new or struggling colleagues can enhance community and belonging. Recognizing and celebrating teachers’ achievements through regular awards or public acknowledgment can also boost morale and job satisfaction. Qualitative findings revealed that collegial support acts as a buffer against burnout, making structured peer mentoring and recognition programs particularly impactful when embedded within a generally supportive climate.

Addressing challenging job demands is equally critical. Institutions should regularly assess workloads —including teaching, research, and administrative duties— to prevent teacher exhaustion and implement clear policies aimed at establishing realistic expectations and limiting excessive burdens. Flexible scheduling options, where feasible within the institutional context, could alleviate workload pressures. Providing adequate resources, such as teaching assistants, updated technology, and sufficient professional development funds, is essential to help teachers manage their responsibilities more effectively. Participants highlighted the stress caused by unrealistic workloads and the pressure to prioritize research over teaching. Addressing these systemic stressors through transparent workload models and equitable evaluation criteria is crucial for enhancing teacher well-being.

Supportive leadership and transparent communication are also vital for creating a positive work environment. School leaders should actively foster approachability, maintain open-door policies, and encourage teachers to voice their concerns and suggestions without fear of retribution. Establishing regular forums for teacher input into relevant policy and curriculum decisions can foster a collaborative atmosphere. Qualitative evidence reinforced the importance of clear communication and participatory decision-making in reducing feelings of frustration and isolation among teachers.

Emotion regulation training can be a valuable component of professional development programs when offered as a supportive resource rather than an additional requirement. Modules on mindfulness, cognitive reappraisal, and stress management techniques can help teachers develop skills to navigate emotional challenges effectively. Schools could also explore providing access to confidential counseling or coaching services. Peer support groups can further enhance emotional resilience, creating a safe space for teachers to share experiences and coping strategies. Participants identified emotion regulation as a crucial personal tool for managing the emotional toll of teaching, validating the need for institutionalized support that empowers teachers in this area.

This study has limitations that should be acknowledged. Firstly, the reliance on self-report questionnaires for all key variables raises the possibility of common method variance (CMV), which might inflate or deflate the observed relationships between constructs. While participants’ perceptions are central to understanding experiences like perceived climate and burnout, relying solely on this data source means these views are not triangulated against other perspectives (e.g., colleagues, administrators) or more objective indicators (e.g., workload records, observational data where appropriate). Future research should incorporate multi-source data collection (e.g., aggregating team-level climate perceptions, using institutional data on workloads) and potentially multi-method designs (e.g., incorporating classroom observations or physiological stress measures) to mitigate CMV concerns and provide a more comprehensive picture. Secondly, the focus on Chinese EFL university instructors may limit generalizability. Replicating the study with diverse teacher samples (different disciplines, educational levels, cultural contexts) could provide a broader understanding. Thirdly, the cross-sectional design restricts inferences about causality. Longitudinal or experimental/intervention designs are recommended for future research to establish causal links and track the development of burnout over time. Fourthly, the study did not include all potential variables influencing teacher well-being, such as specific personality traits (e.g., neuroticism, resilience), detailed social support networks, or broader societal factors impacting educators in China. Future research could examine these factors for a more holistic view. Fifthly, while measurement invariance across gender was established in this study, warranting comparisons between male and female instructors on these constructs, invariance testing was not extended to other potentially relevant subgroups within the sample. Given that the sample included instructors from public and private universities across different institutional tiers, demonstrating measurement invariance across these institutional characteristics would further strengthen confidence in the model’s applicability throughout the diverse landscape of Chinese higher education. Future research with sufficient subgroup sample sizes is needed to explicitly test measurement invariance across these institutional factors. Lastly, while this study employed a mixed-methods design where qualitative data primarily served to contextualize and enrich quantitative findings (an explanatory function), future research could utilize different mixed-methods approaches for potentially deeper integration or different research goals. For example, an exploratory sequential design (QUAL-> quan) might use initial qualitative work to develop culturally specific measures of climate or demands relevant to Chinese EFL instructors. Alternatively, more advanced integration techniques during analysis, such as quantifying qualitative data or using joint displays, could offer further insights into the interplay between the quantitative patterns and the lived experiences. Addressing these limitations can enhance our understanding of teacher burnout and well-being.

## Data Availability

The data analyzed in this study is subject to the following licenses/restrictions: the datasets analyzed during the current study are available upon reasonable request to the corresponding author. Requests to access these datasets should be directed to Lvliang Li, E-mail: elsalynn@126.com.
